# Supplementary material for: Zebrafish pigment cells develop directly from persistent highly multipotent progenitors
Source: Nat Commun. 2023 Mar 6;14:1258. doi: 10.1038/s41467-023-36876-4 (PMC9988989; doi:10.1038/s41467-023-36876-4)
Supplement: Supplementary file 3 — Description of Additional Supplementary Files [file 41467_2023_36876_MOESM3_ESM.pdf]

Description of Additional Supplementary Files

Supplementary Dataset 1: List of neural crest-related genes assessed by NanoString. In addition, we assessed the housekeeping gene ribosomal protein L13 (rpl13), and also glyceraldehyde-3-phosphate dehydrogenase (gapdh), although the latter proved highly variable between cell-types and was discarded. Data from ZFIN, https://zfin.org/ and from Higdon et al. (2013) quantitative profiling of melanocytes and iridophores re-expressed as digital expression.

Expression in different axial positions at 24 hpf is scored for premigratory NCCs i.e. above NT Key: 0=no expression shown; 1=expression shown, '-' = data not available for that gene at that stage.

Supplementary Dataset 2: MTE Primers

Supplementary Dataset 3: TaqMan primers

Supplementary Dataset 4: TaqMan raw data
