# Supplementary material for: Zebrafish pigment cells develop directly from persistent highly multipotent progenitors
Source: Nat Commun. 2023 Mar 6;14:1258. doi: 10.1038/s41467-023-36876-4 (PMC9988989; doi:10.1038/s41467-023-36876-4)
Supplement: Supplementary file 8 — Reporting Summary [file 41467_2023_36876_MOESM8_ESM.pdf]

Corresponding author(s): Robert Kelsh

Last updated by author(s): Jan 3, 2023

Reporting Summary

Nature Portfolio wishes to improve the reproducibility of the work that we publish. This form provides structure for consistency and transparency in reporting. For further information on Nature Portfolio policies, see our Editorial Policies and the Editorial Policy Checklist.

Statistics

For all statistical analyses, confirm that the following items are present in the figure legend, table legend, main text, or Methods section.

n/a	Confirmed	
☐	☒	The exact sample size (*n*) for each experimental group/condition, given as a discrete number and unit of measurement
☐	☒	A statement on whether measurements were taken from distinct samples or whether the same sample was measured repeatedly
☐	☒	The statistical test(s) used AND whether they are one- or two-sided<br>*Only common tests should be described solely by name; describe more complex techniques in the Methods section.*
☒	☐	A description of all covariates tested
☐	☒	A description of any assumptions or corrections, such as tests of normality and adjustment for multiple comparisons
☐	☒	A full description of the statistical parameters including central tendency (e.g. means) or other basic estimates (e.g. regression coefficient) AND variation (e.g. standard deviation) or associated estimates of uncertainty (e.g. confidence intervals)
☒	☐	For null hypothesis testing, the test statistic (e.g. *F*, *t*, *r*) with confidence intervals, effect sizes, degrees of freedom and *P* value noted<br>*Give P values as exact values whenever suitable.*
☒	☐	For Bayesian analysis, information on the choice of priors and Markov chain Monte Carlo settings
☒	☐	For hierarchical and complex designs, identification of the appropriate level for tests and full reporting of outcomes
☒	☐	Estimates of effect sizes (e.g. Cohen's *d*, Pearson's *r*), indicating how they were calculated

Our web collection on statistics for biologists contains articles on many of the points above.

Software and code

Policy information about availability of computer code

Data collection	Software used to obtain single cell data are described fully in the supplementary methods
Data analysis	Code Availability Statement<br>Computer code is available in Zenodo with the identifier DOI: 10.5281/zenodo.7576584 and distributed under MIT licence. The code has been verified for its compliance with guidelines in Nature Code and Software submission checklist. Particularly this version of the code runs at MacBook Pro (13-inch, Mid 2012, 2.5 GHz Intel Core i5, 16 GB 1600 MHz DDR3) running MacOS 10.12.6. Scripting was done for R console ver. 3.5.2, using Seurat 2.3.4, slingshot 1.0.0, and tidyverse 1.2.1 with ggplot2 3.3.3. Published single cell data integration was conducted on the server with 128 cores (Intel Core I5 processors) and 512 Gb of RAM running 18.04.1-Ubuntu Linux 5.4.0-122-generic kernel. Batch correction and data integration scripts was developed on R 4.1.1 version using batchelor 1.14.0 package.

For manuscripts utilizing custom algorithms or software that are central to the research but not yet described in published literature, software must be made available to editors and reviewers. We strongly encourage code deposition in a community repository (e.g. GitHub). See the Nature Portfolio guidelines for submitting code & software for further information.

Data

Policy information about availability of data

All manuscripts must include a data availability statement. This statement should provide the following information, where applicable:

- Accession codes, unique identifiers, or web links for publicly available datasets
- A description of any restrictions on data availability
- For clinical datasets or third party data, please ensure that the statement adheres to our policy

The Nanostring nCounter raw data and TaqMan assay data have been deposited in the National Center for Biotechnology Information Gene Expression Omnibus (GEO) and are accessible through the GEO Series accession number GSE185592.

Human research participants

Policy information about studies involving human research participants and Sex and Gender in Research.

Reporting on sex and gender	No human research participants were involved
Population characteristics	n/a
Recruitment	n/a
Ethics oversight	n/a

Note that full information on the approval of the study protocol must also be provided in the manuscript.

Field-specific reporting

Please select the one below that is the best fit for your research. If you are not sure, read the appropriate sections before making your selection.

☒ Life sciences ☐ Behavioural & social sciences ☐ Ecological, evolutionary & environmental sciences

For a reference copy of the document with all sections, see nature.com/documents/nr-reporting-summary-flat.pdf

Life sciences study design

All studies must disclose on these points even when the disclosure is negative.

Sample size	Sample sizes were limited by practical issues (e.g. labour intensity for fate-mapping; financial constraints for NanoString profiling).
Data exclusions	Nanostring counter data: (i) we removed 3% cells with the top counts of the negative control probes; (ii) we removed all cells with only 3 or less test probes (genes) having counts greater than the selected threshold, only test probes (genes) other than those of internal housekeeping housekeeping (rpl13) or spike-in (kanamycin) controls were considered; (iii) we removed cells with very low probe counts of the internal housekeeping (rpl13) or spike-in (kanamycin) controls (1/50 of the median); (iv) we removed cells with poor norm factors of the NanoStringNorm() function from the NanoStringNorm R package (less than 0.3 or greater than 3) and the estimated background greater than the selected threshold, cormalization was conducted using the sum of probes statistics (kanamycin spike-in and rpl13 internal controls) as the reference housekeeping class with 'mean and 2sd' selected for the background.; (v) we removed data for probes (genes) with reliable expression only in five or less cells.<br>Taqman data: we removed cells with low spike-in control (less than 0.05 quantile) and low total expression (less than 0.05 quantile).
Replication	Experiments were repeated three independent times with equal number of embryos for WT fish. For experiments with mutant fish, the number of embryos varied according to the size of the spawn.
Randomization	Embryos were randomly assigned.
Blinding	Where mutant fish were compared to WT sibling, quantification and analysis was performed prior to genotyping.

Reporting for specific materials, systems and methods

We require information from authors about some types of materials, experimental systems and methods used in many studies. Here, indicate whether each material, system or method listed is relevant to your study. If you are not sure if a list item applies to your research, read the appropriate section before selecting a response.

Materials & experimental systems

n/a	Involved in the study
☐	☒ Antibodies
☒	☐ Eukaryotic cell lines
☒	☐ Palaeontology and archaeology
☐	☒ Animals and other organisms
☒	☐ Clinical data
☒	☐ Dual use research of concern

Methods

n/a	Involved in the study
☒	☐ ChIP-seq
☐	☒ Flow cytometry
☒	☐ MRI-based neuroimaging

Antibodies

Antibodies used

Goat anti Rabbit Alexa Fluor488 (Invitrogen Cat. No. A32731TR)
Rabbit anti GFP (Invitrogen Cat. No. A11122)
Mouse Monoclona anti GFP (AB_221569)
anti-acetylated tubulin AB_477585

Validation

The anti-GFP mAb has been used in 13 publications listed in ZFIN - https://zfin.org/ZDB-ATB-081009-2
The acetylated tubulin Ab is widely used in the zebrafish community, with 535 publications - see https://zfin.org/search?category=Antibody&q=acetylated.

Samples with and without GFP expression where stained with and without primary and secondary antibody to verify specificity of antibody detection.
Rabbit anti GFP (Invitrogen Cat. No. A11122) Primary antibody used in 1588 publication
Goat anti Rabbit Alexa Fluor488 (Invitrogen Cat. No. A32731TR) Secondary antibody used in 668 References

Animals and other research organisms

Policy information about studies involving animals; ARRIVE guidelines recommended for reporting animal research, and Sex and Gender in Research

Laboratory animals	Zebrafish (Danio rerio), AB strain
Wild animals	N/A
Reporting on sex	All data refer to embryos/early larvae prior to a stage when sex determination has occurred.
Field-collected samples	No samples collected from field
Ethics oversight	Approved by Home Office and University of Bath Ethics Committee

Note that full information on the approval of the study protocol must also be provided in the manuscript.

Flow Cytometry

Plots

Confirm that:

☒ The axis labels state the marker and fluorochrome used (e.g. CD4-FITC).

☒ The axis scales are clearly visible. Include numbers along axes only for bottom left plot of group (a 'group' is an analysis of identical markers).

☒ All plots are contour plots with outliers or pseudocolor plots.

☒ A numerical value for number of cells or percentage (with statistics) is provided.

Methodology

Sample preparation

Embryos were incubated up to the desired stage (from 14 to 72 hpf) in standard embryo media at 29 degrees celcius. To prevent melanisation in embryo melanocytes, PTU (N-Phenylthiourea, Cat# 7629, Sigma-Aldrich) was added at a final concentration of 0.003% at 24 hpf. eGFP expression was heat-shock induced at 42oC for 1 hpur. Dechorionated embryos where digested using TrypLE™ Express Enzyme (Cat#12605036, ThermoFisher Scientific) in ratio of 10 ml per 100 embryos, containing 0.003% Tricaine for 30-90 minutes at 37o C in agitation. Manual dissociation of tissue was performed using a Pasteur pipette (10-15 times). Undigested fragment where removed by filtering the cell suspension through a100-micron strainer (MACS SmartStrainers, Cat# 130-098-463, Miltenyi Biotech.). The filtered cell suspension was centrifuged for 5 min at 500xg at 4o C. The cell pellet was re-suspended in DPBS and filtered a second time through 30-micron strainer (MACS SmartStrainers, Cat# 130-110-915, Miltenyi Biotech.). The filtered cell suspension was centrifuged a second time using the

	conditions. The cell pellet was re-suspended in 0.5-1 ml of cell media (2% FCS, DPBS:HBSS=1:1 and 1 M SYTOX Blue Dead Cell stain (ThermoFisher Scientific) for cell sorting.
Instrument	BD FACSAria III cell sorter
Software	BD FACSDiva software
Cell population abundance	7-9% of population 1 as described below.
Gating strategy	Total events were visualized using a FSC-A vs SSC scatter plot from which the main Population 1 (P1) was gated to discard fragmented cells. Exclusion of double event was performed using a FSC-A vs FSC-W scatter plot from which population 2 (P2) was gated. Fluorescence (GFP) and viability (DAPI) was assessed using a two parameter scatter plot.

☒ Tick this box to confirm that a figure exemplifying the gating strategy is provided in the Supplementary Information.
