# Supplementary material for: Zebrafish pigment cells develop directly from persistent highly multipotent progenitors
Source: Nat Commun. 2023 Mar 6;14:1258. doi: 10.1038/s41467-023-36876-4 (PMC9988989; doi:10.1038/s41467-023-36876-4)
Supplement: Supplementary file 1 — Supplementary Information [file 41467_2023_36876_MOESM1_ESM.pdf]

Supplementary Information

Zebrafish pigment cells develop directly from persistent highly multipotent progenitors

Tatiana Subkhankulova[1], Karen Camargo Sosa[1], Leonid A. Uroshlev[2], Masataka Nikaido[1,a], Noah Shriever[1], Artem S. Kasianov[2,3,4], Xueyan Yang[1,b], Frederico S. L. M. Rodrigues[1], Thomas J. Carney[1,c], Gemma Bavister[1], Hartmut Schwetlick[5], Jonathan H.P. Dawes[5], Andrea Rocco[6,7], Vsevolod Makeev[2,3,8] and Robert N. Kelsh*[,1]

* E-mail:bssrnk@bath.ac.uk

These authors contributed equally: Tatiana Subkhankulova and Karen Camargo Sosa

Supplementary Tables

Supplementary Table 1. Key data for scRNA-seq studies used in meta-analysis.

GEO ID	Authors	PMID	Cell marking	Sequencing depth, reads/cell	Number of cells
GSE112294 https://www.ncbi.nlm.nih.gov/geo/query/acc.cgi?acc=GSE112294	Wagner, DE et al., 2018[1]	29700229	*ß-actin2-sfGFP*	~20000	26785
GSE131136 https://www.ncbi.nlm.nih.gov/geo/query/acc.cgi?acc=gse131136	Saunders, LM et al., 2019[2]	31140974	*Tg(nVenus-2a-nfnB) sox10:Cre:mCherry+*	~40000	28125
GSE152906 https://www.ncbi.nlm.nih.gov/geo/query/acc.cgi?acc=GSE152906	Howard, AG et al., 2021[3]	33591267	*Tg(−4.9sox10:GFP)ba2Tg*	~74000 for 48hpf, ~62000 for 69 hpf	4880
GSE163907 https://www.ncbi.nlm.nih.gov/geo/query/acc.cgi?acc=GSE163907	Lencer, E et al., 2021[4]	34397384	*Tg(sox10:eGFP)ba2, Tg(sox10:TagRFP)co26TG*	~160000	607
GSE178364 https://www.ncbi.nlm.nih.gov/geo/query/acc.cgi?acc=GSE178364	Brombin, A et al., 2022[5]	35021087	*Tg(crestin:mCherry; mitfa:GFP)*	~50000	1519
GSE190610 https://www.ncbi.nlm.nih.gov/geo/query/acc.cgi?ac	Kenny, C et al., 2022[6]	35580127	Tg(mitfa-GFP)	~5300	12106

c=GSE190610					
**Total cells**					74022

Supplementary Table 2. Inactive version of NPM-Ltk has minimal activity to induce precocious/ectopic iridophores.

	No. of embryos injected	Normal morphology		Dead or deformed
		+ectopic	No additional iridophores	
Tg(sox10:*NPM-ltk(DK))*	327 (100%)	6 (1.8%)	240(73.4%)	81(24.8%)
Tg(sox10:*NPM-ltk*)	323 (100%)	69 (21.4%)	170(52.6%)	84(26.0%)

Notes :

"+ectopic" here includes embryos with precocious and/or ectopic iridophores. Deformed means serious defects in body axis, making it difficult to identify iridophores in normal position.

Supplementary Table 3. Single cell Nanostring hybridisation profiling

Cell type	Tails	*sox10-*	IP	MC	Regular WT							
Stage, hpf	24	30	72	72	18	21	24	30	36	48	60	72
#cells	108	135	25	19	46	37	54	53	65	43	80	66

Supplementary Figures

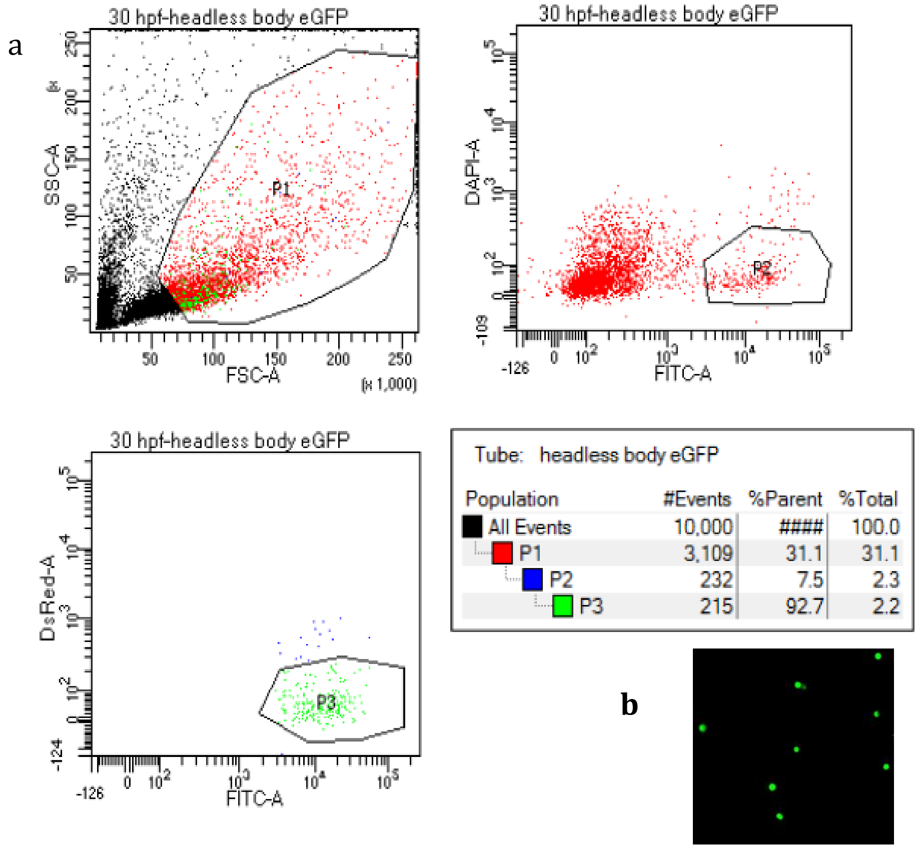

Population	#Events	%Parent	%Total
All Events	10,000	####	100.0
P1	3,109	31.1	31.1
P2	232	7.5	2.3
P3	215	92.7	2.2

Supplementary Figure 1 Isolation of GFP-positive (Sox10-positive) neural crest cells

36 hpf stage used as an example.

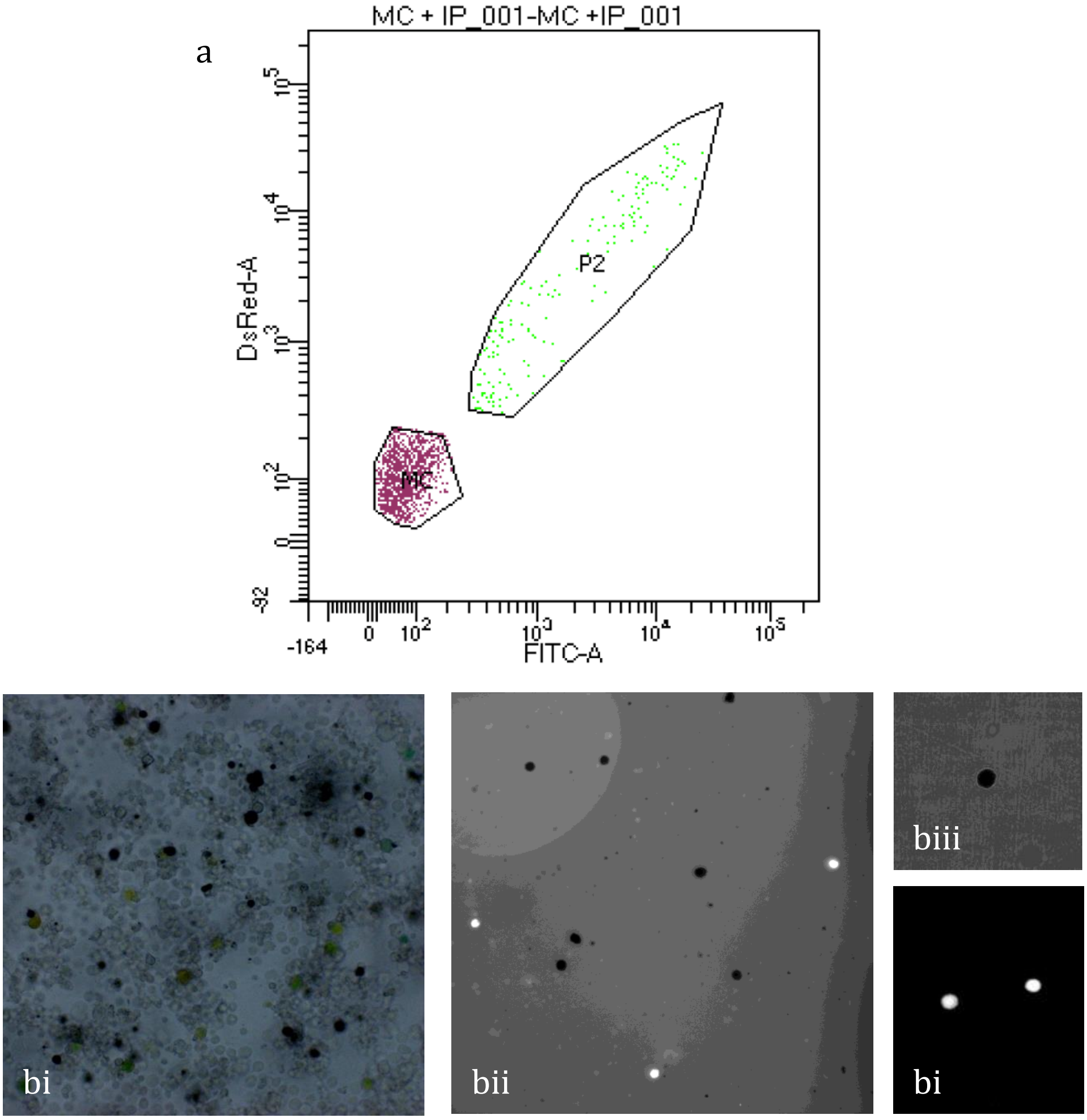

Supplementary Figure 2 Isolation of control melanocytes and iridophores from 72 hpf wild-type zebrafish.

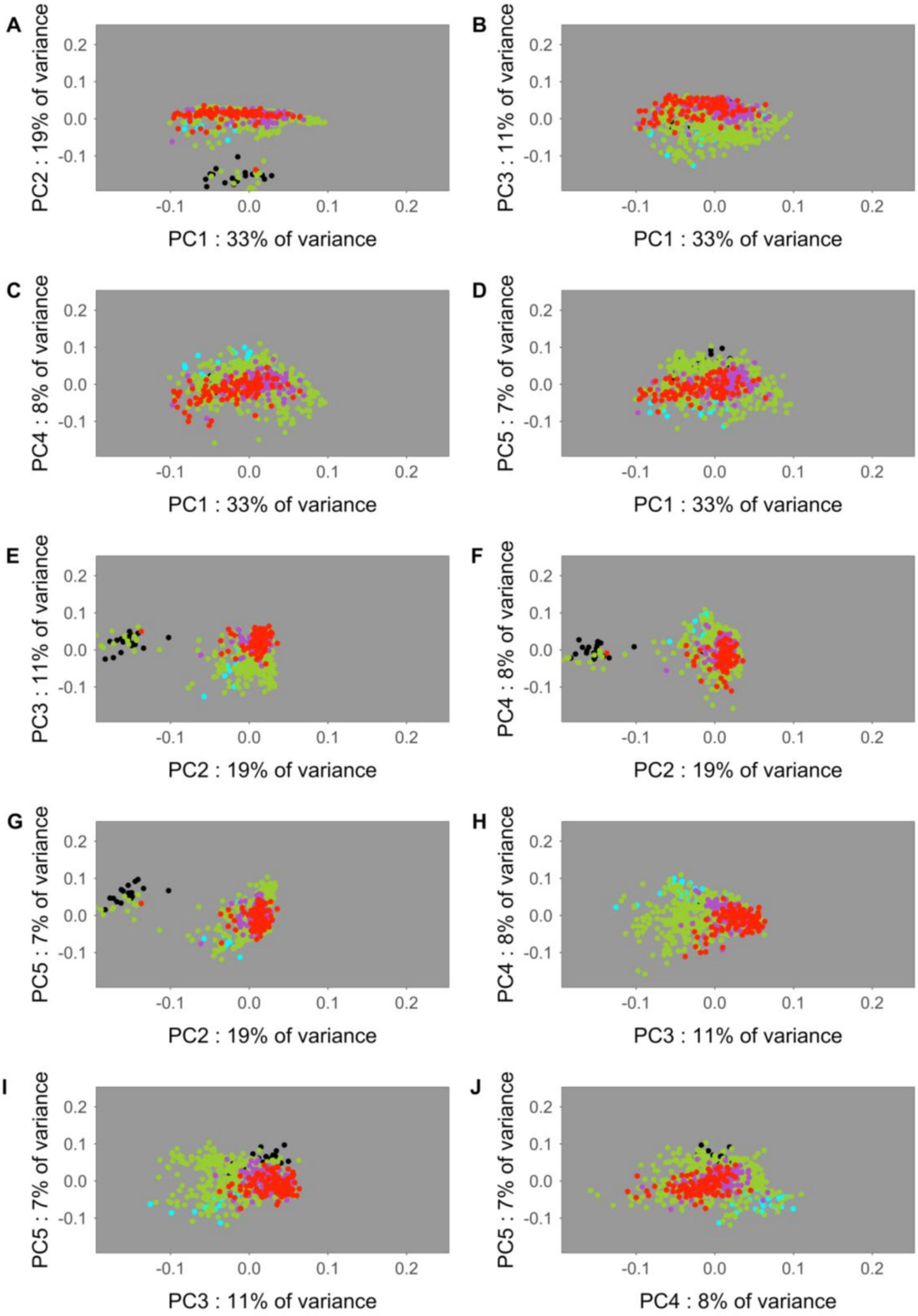

Supplementary Figure 3. Projection of cells distributions on different pairs of principal components. Colors: (green) Regular WT cells, all hpf pooled; (red) cells taken from fish tails; (cyan) control iridophores; (black) control melanocytes; (magenta) sox10 mutants.

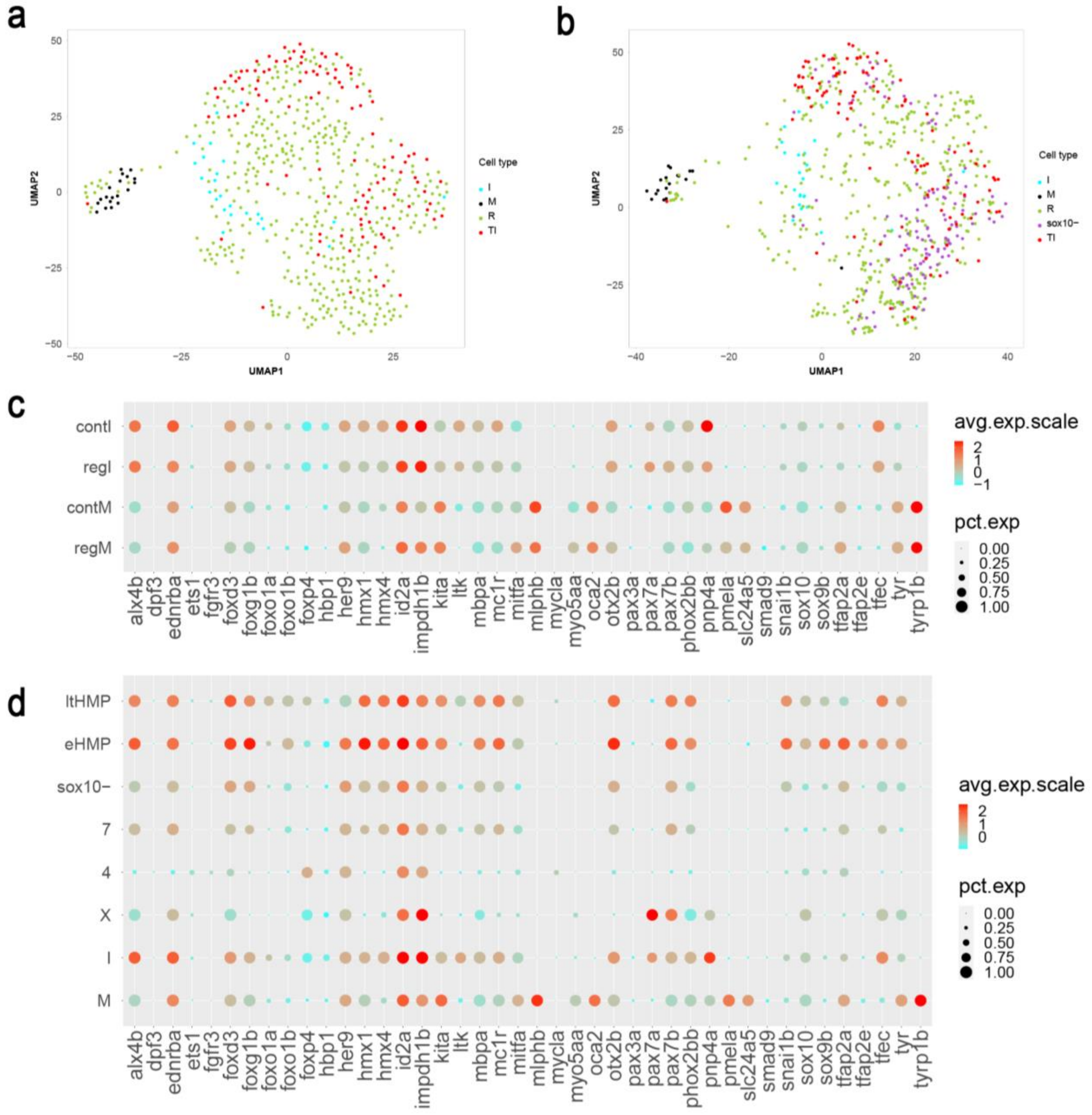

Supplementary Figure 4. Distribution of control cells across clusters. a, Control melanocytes (M, black), control iridophores (I, cyan) and tail cells (24 hpf)(TI, red), and regular WT cells (R, green) plotted on 2D UMAP projection . **b**, 2D UMAP projection shows that *sox10* mutants (sox10-; purple) occupy a region overlapping with some of the WT highly multipotent cells (the area enriched with TI cells, both eHMP and ltHMP clusters from Figure 2 are relevant). **c**, Dot plot comparison of gene expression profiles of control pigment cells and regular cells found in the same clusters. Melanocytes: control cells (contM) and others from FACS-sorted WT population (regM). Iridophores: control cells (contI) and others from

FACS-sorted WT population (expI). Highly expressed genes in each cell-type match expectations from published literature. **d** Dot plot of gene expression profile of WT clusters (see Fig. 2c) and *sox10* mutant cells. Note that low expression of *ltk* in *sox10* mutants in the dot plot comes out of combined imputation and normalization conditions (compare Fig. 2e showing that 52% of cells show detectable *ltk*). Expression colour scheme corresponds to expression magnitude span of all genes and is not gene specific. The source data, after quality control and imputation, for the single cell diagrams in Supplementary Fig. 3-9 and 14 is contained in the Source Data file.

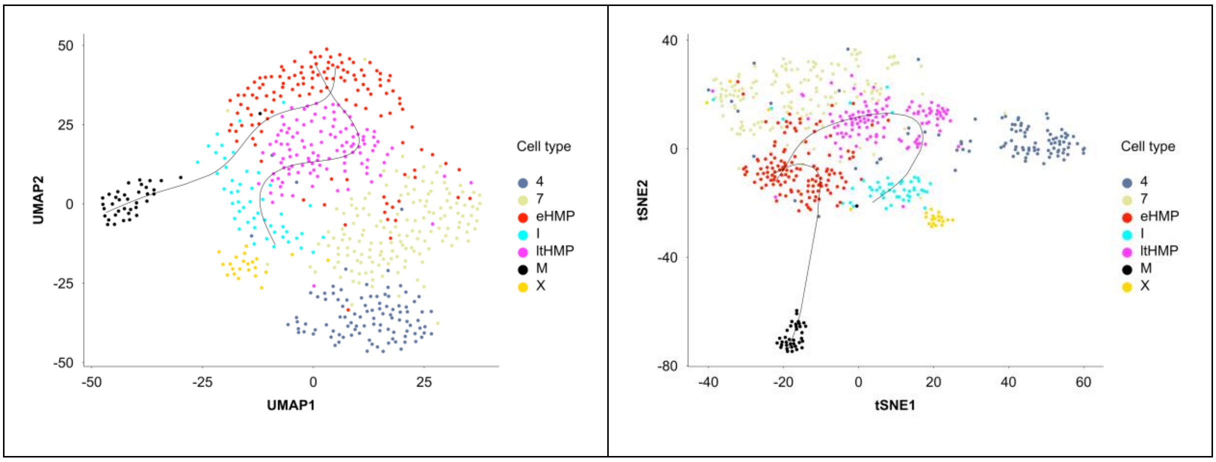

Supplementary Figure 5. Cell clustering and pseudotime curves in UMAP and tSNE planes.

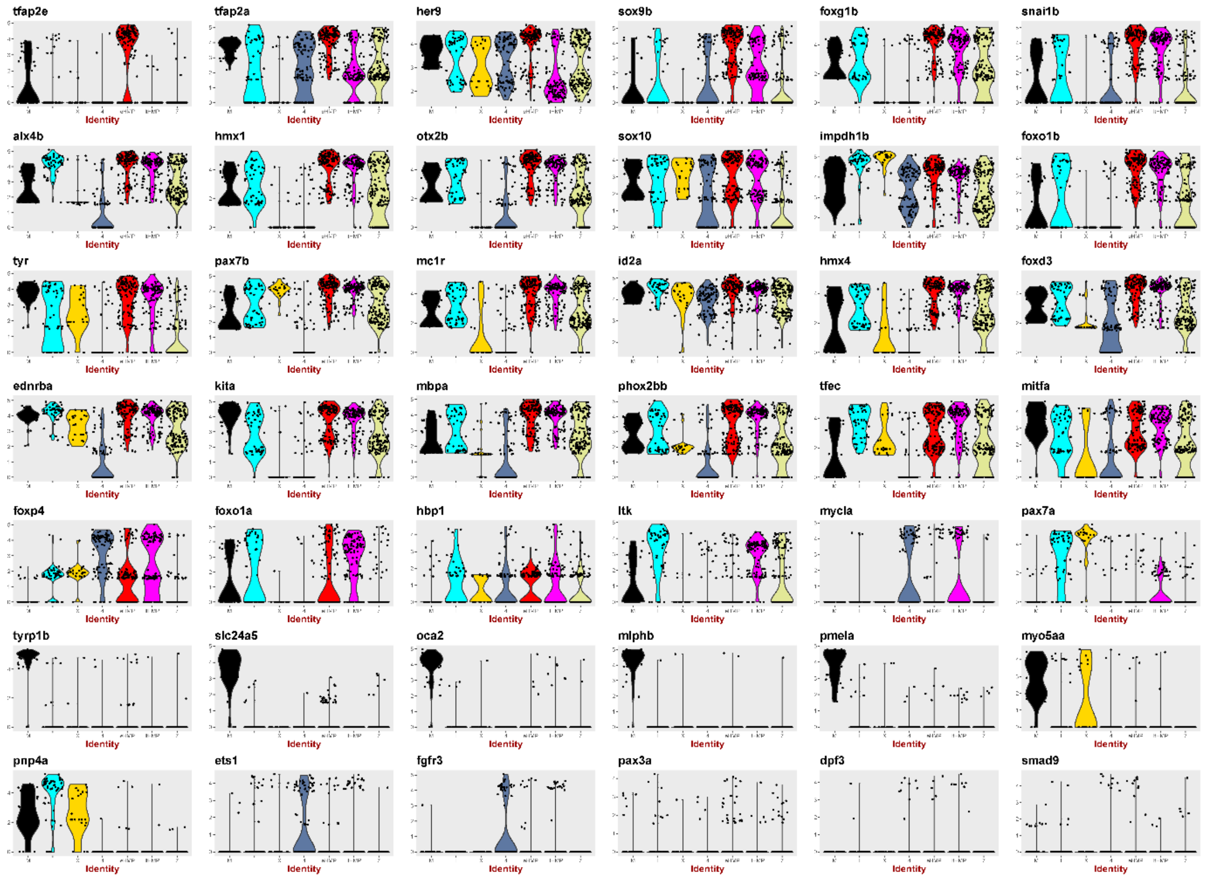

Supplementary Figure 6 Gene expression profiles of WT cell clusters (see Fig. 2c).

Violin plots of expression levels of each gene in each WT cell.

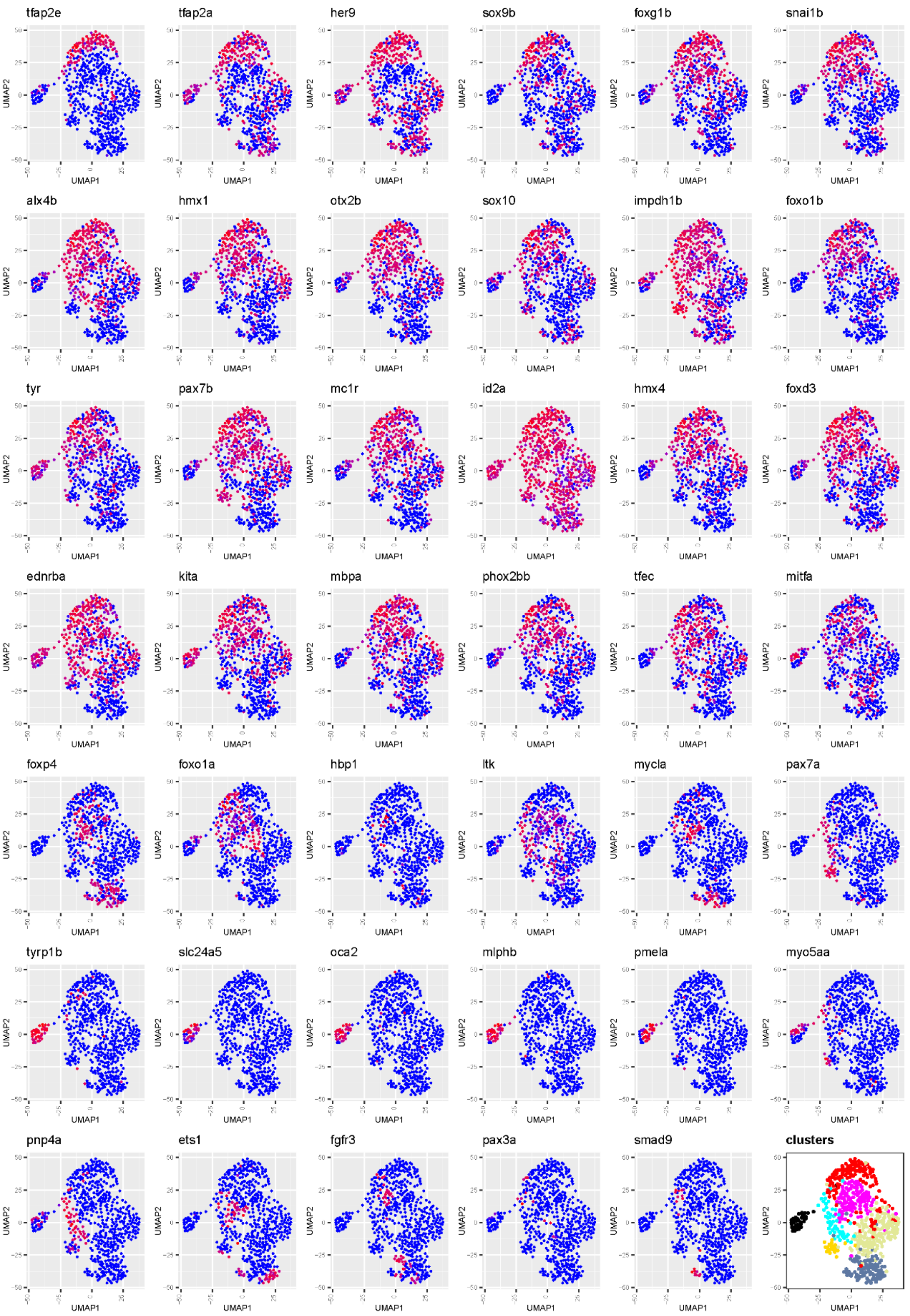

Supplementary Figure 7 Gene expression profiles of WT cell clusters. Feature plots show regionally localized gene expression in different areas of 2D UMAP plane. Early neural

crest specification markers (*sox9b* and *snail2,* but also *tfap2a* and *tfap2e*) are expressed in the cells found in the top right corner of the plane (eHMP (red) and ltHMP (pink) clusters in bottom right reproduction of Fig. 2c). Other genes like *ltk* and *foxo1a* are expressed more centrally (ltHMP). Note how eHMP and ltHMP clusters express genes with known role in fate specification of all pigment cell (*mitfa, pax7b, tfec, ltk)*, neuronal (*phox2bb)* and glial (*sox10*) cell-types, whereas no clusters show specific combinations consistent with MIXG (*mitfa, pax7b, tfec, ltk, sox10,* but not *phox2bb*) nor MI (*mitfa, tfec, ltk,* but not *pax7b* or *phox2bb*) intermediates. Specific markers of differentiating melanocytes (including *pmel, mlphb* and *oca2*) readily identify the discrete melanocyte cluster on the left. The iridophore cluster is less distinctive due to the known early progenitor expression of many key iridophore specification genes, but lies to the left of the centre in these plots and shows concentrated expression of *pnp4a, ltk, tfec, ednrba.* This analysis also confirms our recent demonstration from whole mount in situ hybridization studies that *pnp4a* is widespread in pigment cells[7]. Bottom right panel shows clusters. The identity of cluster 4 is less clear, but in the zebrafish RNA-Seq data the intersecting area of the genes defining this cluster (*foxp4, her9, id2a,* and *impdh1b,* with some expression of the early *tfap2a* gene) more or less overlaps with the area of expression of *twist1a*, so that at least some of these cells may include skeletogenic fates.

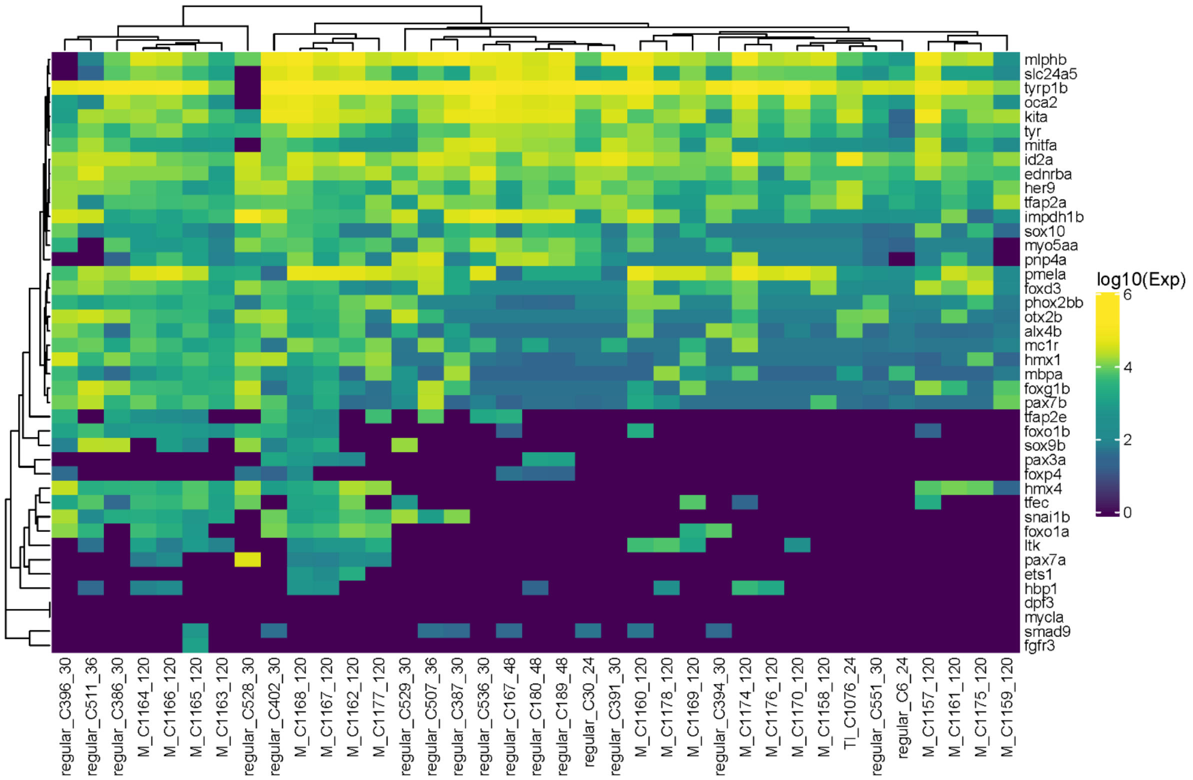

Supplementary Figure 8 Melanocyte cluster contains distinct sub-clusters. Expression profiles of melanocytes shows distinct sub-clusters; cells in right clusters show detectable expression of genes (e.g. *tfec, ltk, sox9b, snail2*) associated with earlier stages*.* Cell labels distinguish control melanocytes (labels begin ‘M_’) from those identified from regular WT cells (labels begin ‘regular_’) and from Tail cells (labels begin ‘TI_’).

a

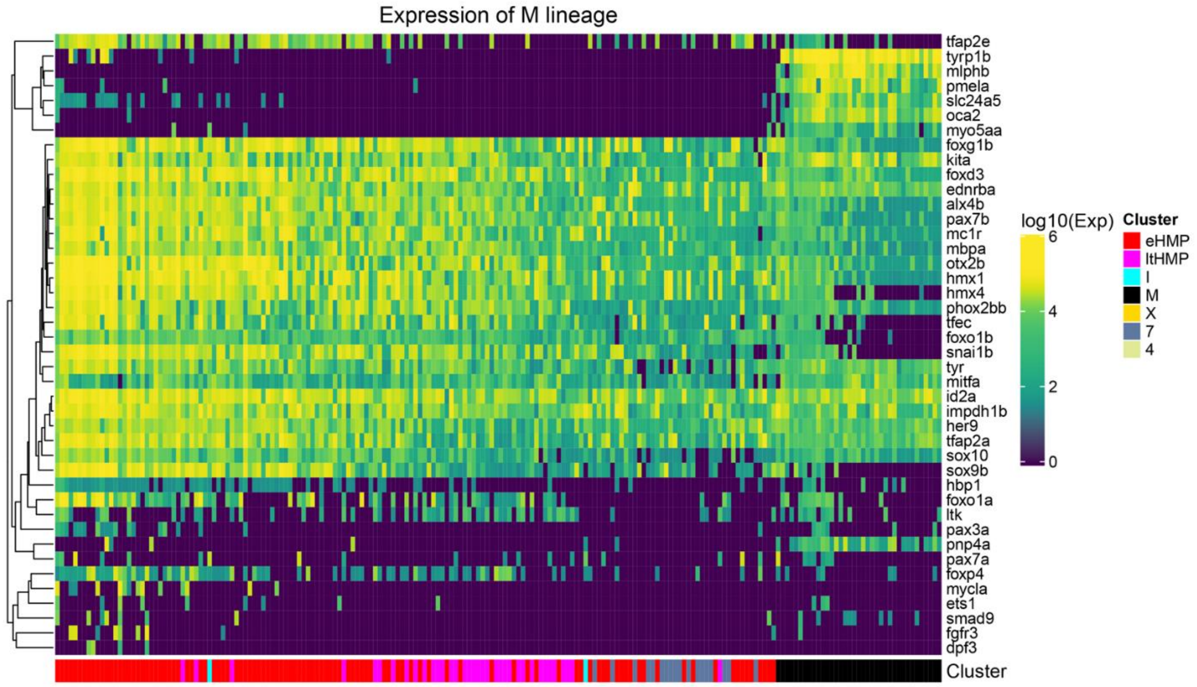

Expression of M lineage
tfap2e
tyrp1b
mlphb
pmela
slc24a5
oca2
myo5aa
foxg1b
kita
foxd3
ednrba
alx4b
pax7b
mc1r
mbpa
otx2b
hmx1
hmx4
phox2bb
tfec
foxo1b
snai1b
tyr
mitfa
id2a
impdh1b
her9
tfap2a
sox10
sox9b
hbp1
foxo1a
ltk
pax3a
pnp4a
pax7a
foxp4
mycla
ets1
smad9
fgfr3
dpf3
Cluster
log10(Exp)
6
4
2
0
Cluster
eHMP
ltHMP
I
M
X
7
4

b

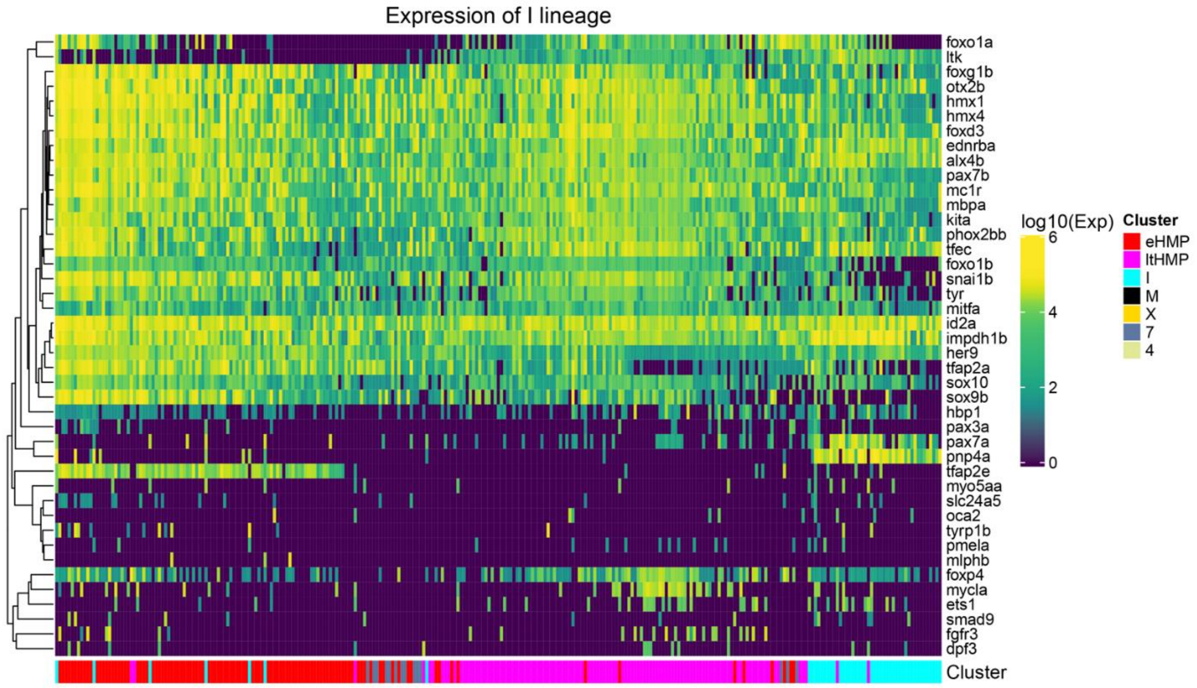

Expression of I lineage
foxo1a
ltk
foxg1b
otx2b
hmx1
hmx4
foxd3
ednrba
alx4b
pax7b
mc1r
mbpa
kita
phox2bb
tfec
foxo1b
snai1b
tyr
mitfa
id2a
impdh1b
her9
tfap2a
sox10
sox9b
hbp1
pax3a
pax7a
pnp4a
tfap2e
myo5aa
slc24a5
oca2
tyrp1b
pmela
mlphb
foxp4
mycla
ets1
smad9
fgfr3
dpf3
Cluster
log10(Exp)
6
4
2
0
Cluster
eHMP
ltHMP
I
M
X
7
4

Supplementary Figure 9 Pseudotemporal analysis of pigment cell differentiation. Heat maps showing pseudotime differentiation trajectories for melanocytes (**a**) and iridophores (**b**). Note that these representations were generated by collecting cells close to the trajectories drawn through the centres of clusters ordered into the cluster lineage tree in 42D space of gene expression. The trajectories (principle curves) were also constructed in 42D gene expression space and used to order cells in the heatmap. The cells in the colour chart below have been coloured according to assignment to particular clusters constructed after dimension reduction with UMAP to 2D. Cells from cluster 7 express a limited number of differentiation markers and their identity is uncertain.

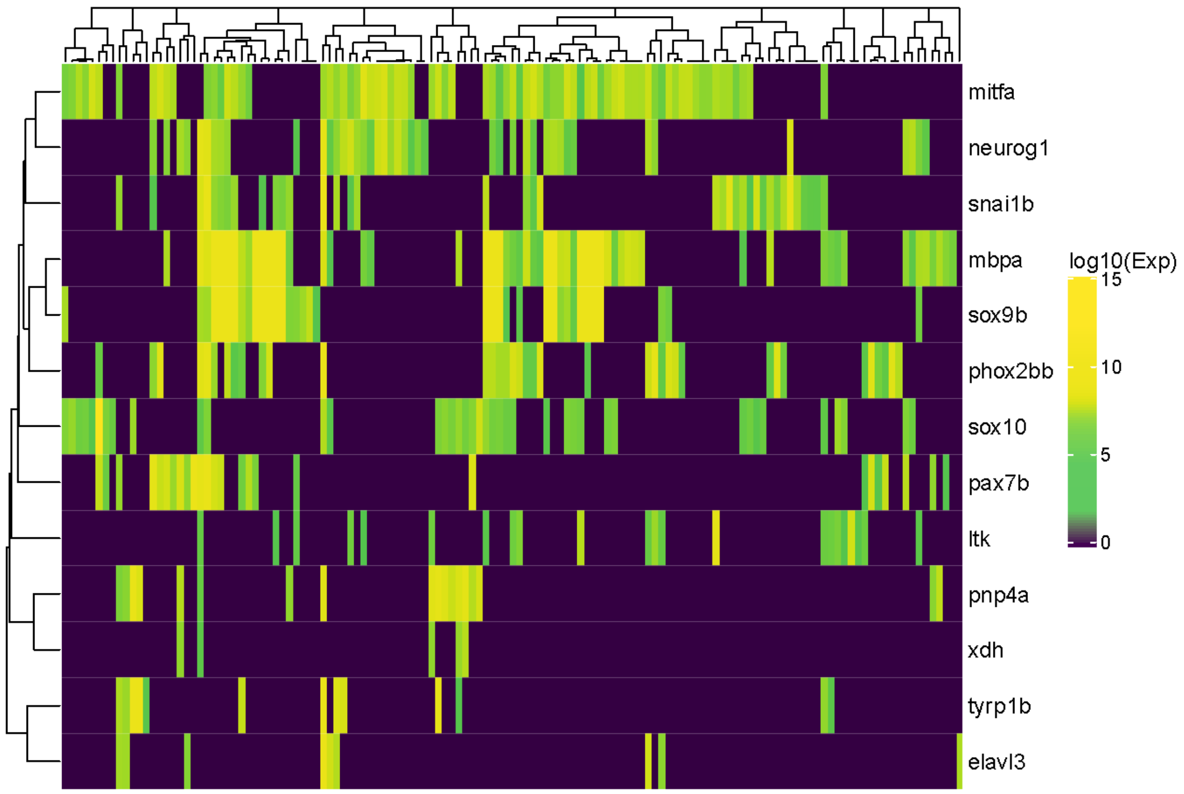

Supplementary Figure 10 Independent TaqMan assay qRT-PCR assessment of overlapping fate specification gene expression in 24 hpf WT embryos. Note that individual cell profiles often show expression of fate specification transcription factor genes (*mitfa, pax7b, tfec, ltk, sox10, phox2bb*), with multiple genes being detected in each individual cell, whilst usually lacking detectable expression of pigment cell and neuronal differentiation markers (*pnp4a, xdh, tyrp1b,elavl3)*. Note also widespread expression of glial differentiation marker *mbpa*. Source data are provided in Source Data file.

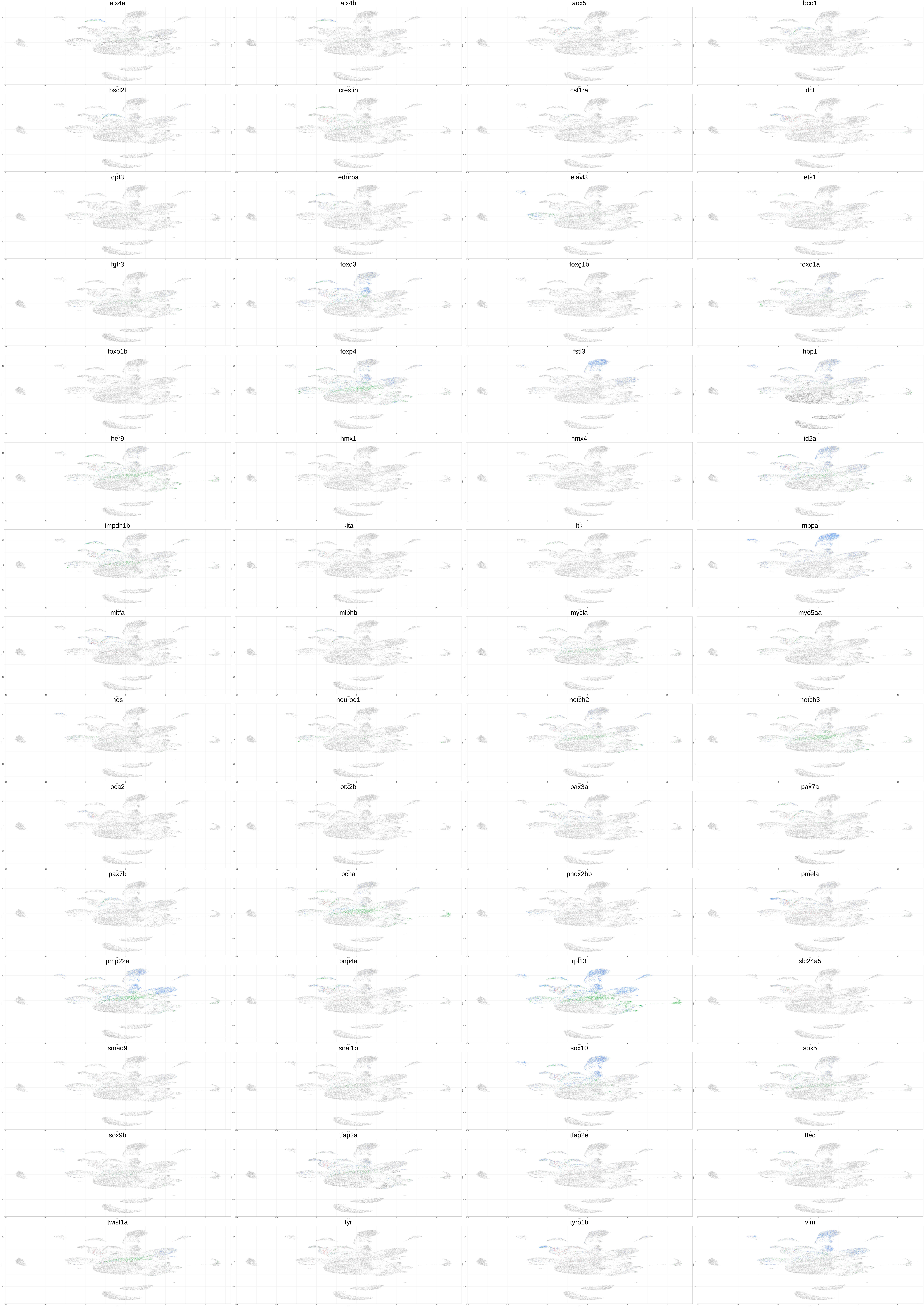
alx4a
alx4b
aox5
bco1
bscl2l
crestin
csf1ra
dct
dpf3
ednrba
elavl3
ets1
fgfr3
foxd3
foxg1b
foxo1a
foxo1b
foxp4
fstl3
hbp1
her9
hmx1
hmx4
id2a
impdh1b
kita
ltk
mbpa
mitfa
mlphb
mycla
myo5aa
nes
neurod1
notch2
notch3
oca2
otx2b
pax3a
pax7a
pax7b
pcna
phox2bb
pmela
pmp22a
pnp4a
rpl13
slc24a5
smad9
snai1b
sox10
sox5
sox9b
tfap2a
tfap2e
tfec
twist1a
tyr
tyrp1b
vim

Supplementary Figure 11 Contribution of different data sources into integrated RNA-Seq data for each gene. UMAP transformation after the batch removal for all cells in Supplementary Table 2. Cells are colored by the study the cell data are taken from:(Orange ●) Brombin, A et al., 2022, GSE178364; (Brown ●) Howard, AG et al., 2021**,** GSE152906; (Green ●) Kenny, C et al., 2022, GSE190610; (Cyan ●) Lencer, E et al., 2021, GSE163907; (Blue ●) Saunders, LM et al., 2019, GSE131136; (Magenta ●) Wagner, DE et al., 2018. Cells with 0 or 1 gene reads are considered as 'not expressing' the target genes and colored grey ●. The data was uploaded from the published sources listed in Supplementary Table 2 and processed as described in the Supplementary Methods section 'Comparison with published RNA-Seq data'

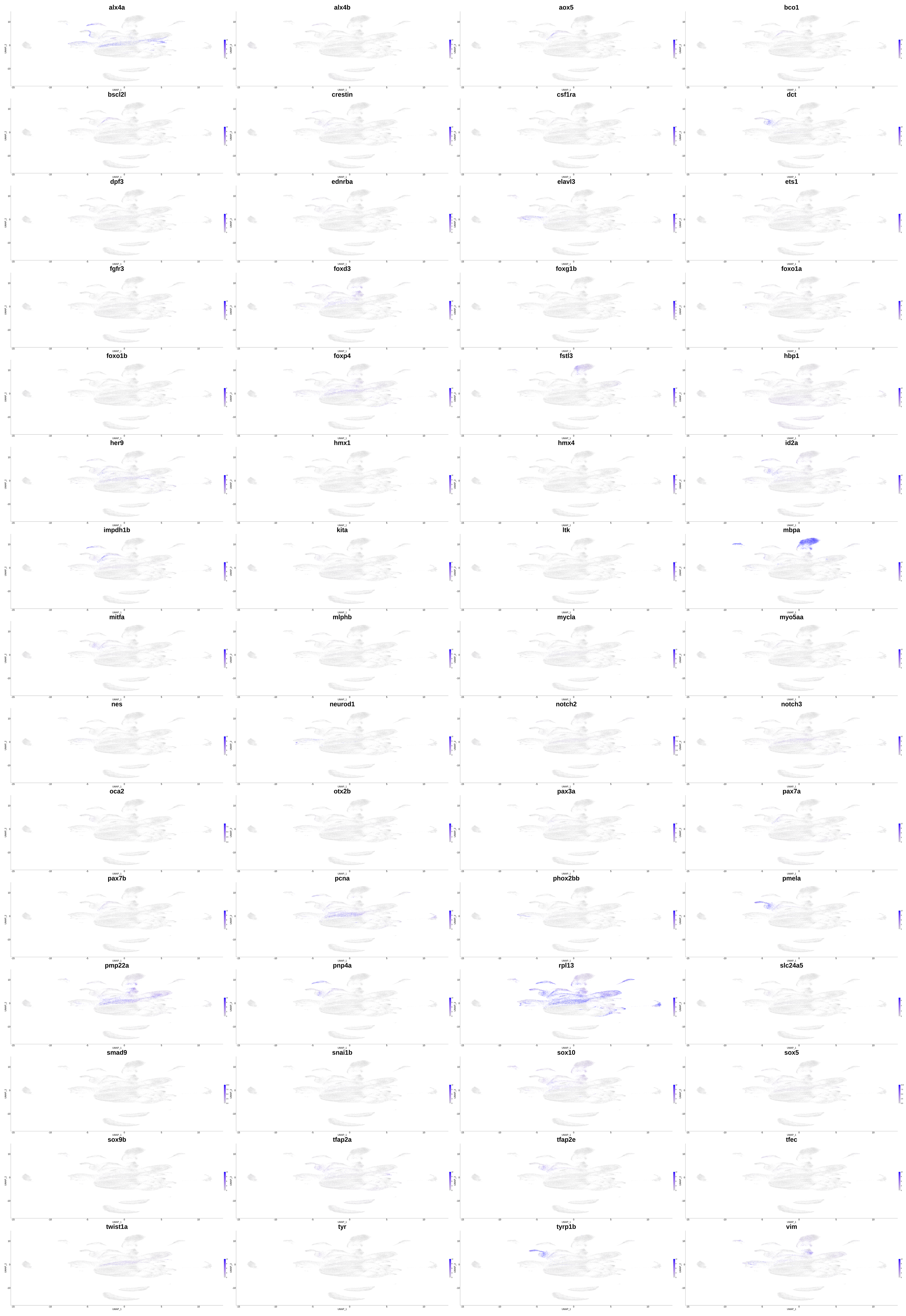
alx4a
alx4b
aox5
bco1
bscl2l
crestin
csf1ra
dct
dpf3
ednrba
elavl3
ets1
fgfr3
foxd3
foxg1b
foxo1a
foxo1b
foxp4
fstl3
hbp1
her9
hmx1
hmx4
id2a
impdh1b
kita
ltk
mbpa
mitfa
mlphb
mycla
myo5aa
nes
neurod1
notch2
notch3
oca2
otx2b
pax3a
pax7a
pax7b
pcna
phox2bb
pmela
pmp22a
pnp4a
rpl13
slc24a5
smad9
snai1b
sox10
sox5
sox9b
tfap2a
tfap2e
tfec
twist1a
tyr
tyrp1b
vim

Supplementary Figure 12 Gene expression profiles in integrated RNA-Seq data. UMAP transformation after the batch removal for all cells in Supplementary Table 2. Cells are colored by the number of gene reads in the cell.

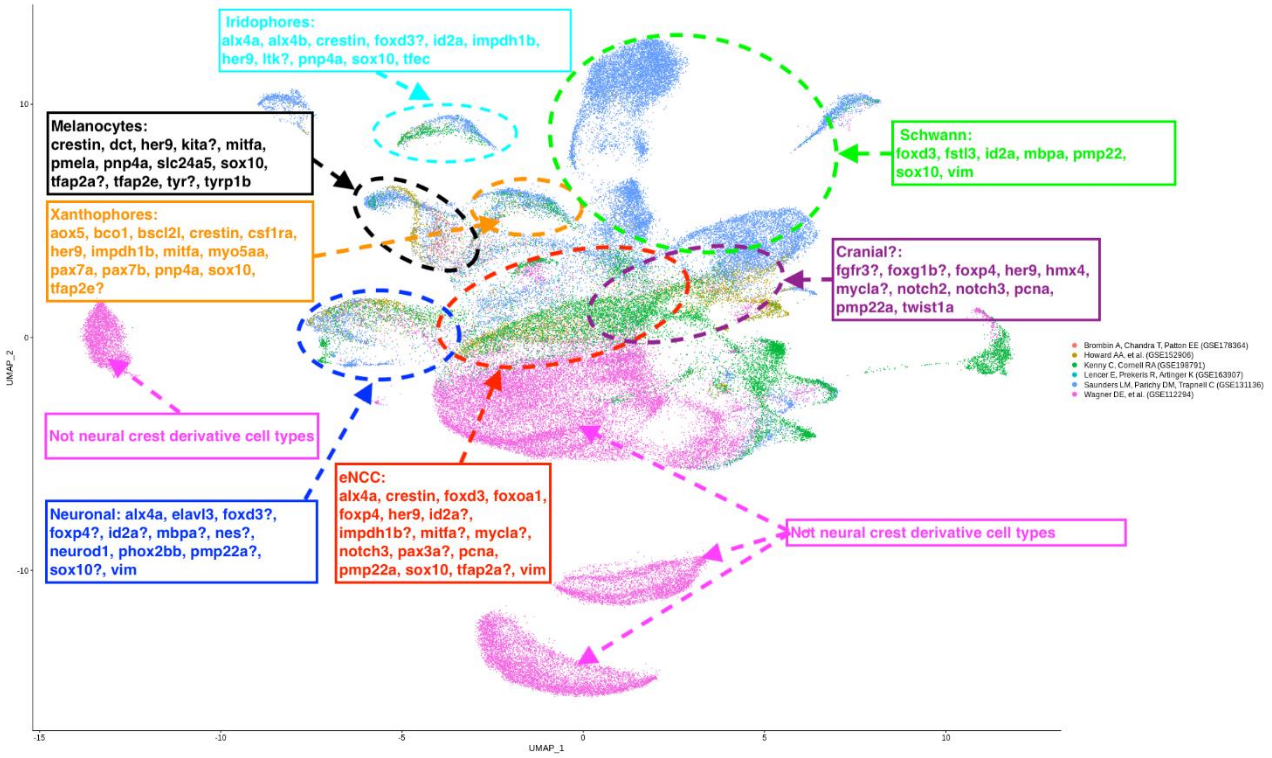

Supplementary Figure 13 Provisional annotation of cell types located in different areas of the UMAP diagram. Annotation is based upon combinations of known gene markers expressed in different NC derivatives.

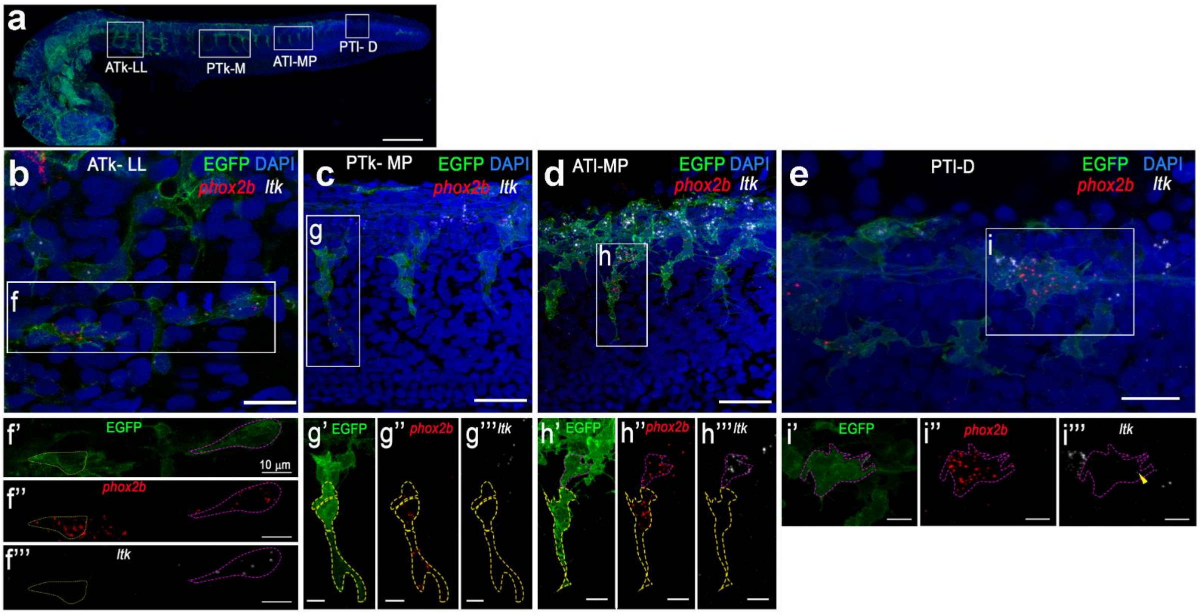

Supplementary Figure 14 Co-expression of *phox2bb* and *ltk in vivo* by RNAscope. Simultaneous immunofluorescent detection of EGFP and RNAscope FISH for *phox2bb* (red) and *ltk* (white) on 24 hpf *Tg*(*sox10:cre; hsp70I:loxp-dsred-STOP-loxp-LYN-EGFP*) embryos after brief heat-shock. **a**, Projection of lateral views shows the lateral line (LL) in the anterior trunk (ATk; **a**), medial pathway (MP) in the posterior trunk (PTk; **b**), medial pathway (MP) in the anterior tail (ATl; **c**) and Dorsolateral (D) Posterior tail (PTl; **d**); NB this is same image as shown on panel **a** in Fig. 2. White boxes indicate close-ups (**b-e**), for which single channel images of EGFP, *phox2bb* and *ltk* for insets boxed in **b-e** are shown in **f'-i'''**. Cell membrane border of EGFP positive cells double-labelled with *phox2bb* and *ltk* are indicated with magenta dashed lines, whereas cell membrane border of *phox2bb* positive cells (lacking *ltk*) are indicated with yellow dashed lines. Scale bars: 200 µm (a), 50 µm (c and d), 20 µm (b and e) and 10 µm (f-i).

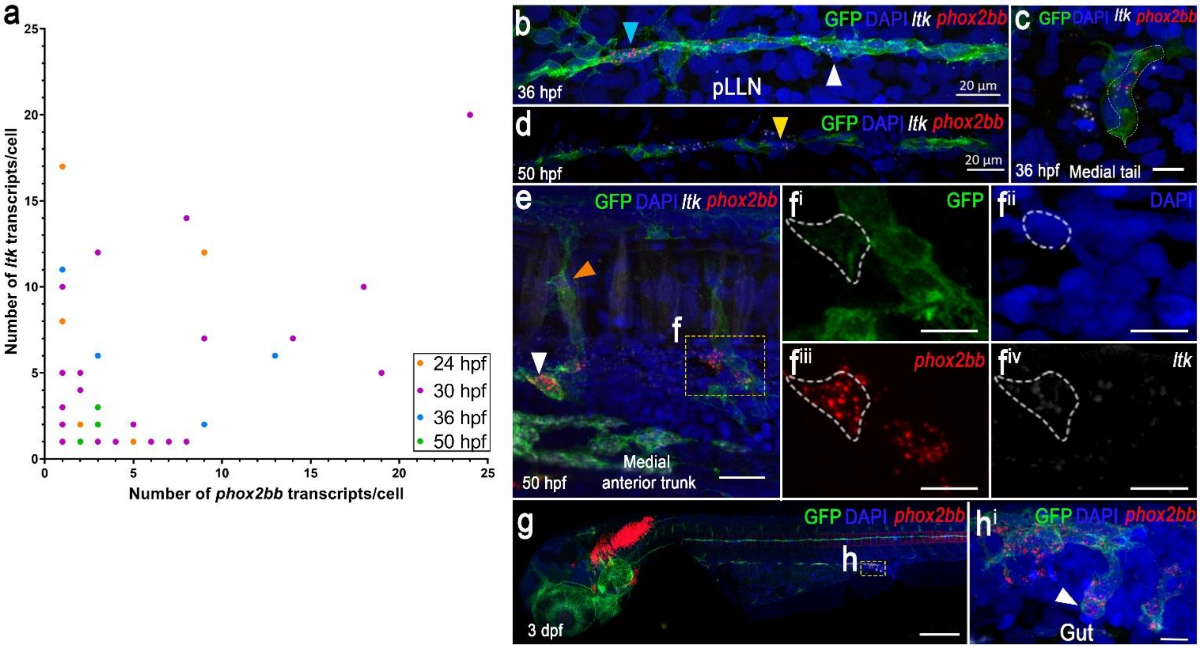

Supplementary Figure 15 Quantitative analysis of the expression of *ltk* and *phox2bb*. Simultaneous immunofluorescent detection of EGFP and RNAscope FISH for *phox2bb* (red) and *ltk* (white) on 24 hpf *Tg*(*sox10:cre; hsp70I:loxp-dsred-STOP-loxp-LYN-EGFP*) embryos after brief heat-shock. **a**, Scatter plot shows distribution of co-expression of ltk and *phoxbb* in single neural crest cells at 24hpf (orange: n=2 embryos), 30 hpf (magenta: n=42 embryos), 36 hpf (blue: n=1 embryos) and 50 hpf (green: n=4 embryos). Quantification was performed on equivalent single fields of view of the tail, posterior trunk and anterior trunk, through the medial and lateral pathways of one half of each embryo. Note that at 50 hpf quantitation was not possible in many cells due to optical limitations caused by the thickness of embryos at this stage; also note that cells expressing just *ltk* or *phox2bb* were not quantitated. **b-h**^{i}, Examples of *phox2bb*-expressing and *phox2bb* and *ltk* co-expressing cells. Single expression of *ltk* (white arrowhead; **b**) and *phox2bb* (light blue arrowhead; **b**) is detected in cells associated with the posterior lateral line nerve primordium (pLLN) at 36 hpf (**b**) and at 50 hpf (**d**). High co-expression of both genes was found on cells located in premigratory positions and in some cells migrating through the medial pathway of the tail at 24 hpf, 30 hpf and 36 hpf (**c**). At 50 hpf cells expressing both genes are restricted to ventral medial pathway (**e**, close-up of boxed region shown in **h**^{i}-**h**^{iv}). At 3 dpf coexpression of *ltk* and *phox2bb* was not detected. At 50 hpf

and 3 dpf *phox2bb* expression was detected in cells associated with DRG (orange arrowhead; **e**), sympathetic neurons (white arrowhead; **e**) and enteric neurons (**i-j**$^{\mathbf{i}}$). Scale bars: 200 μm (g), 20 μm (b, d and e) and 10 μm (c, f^{i}-$f^{V}$and h^{i}). Source data are provided in Source Data file.

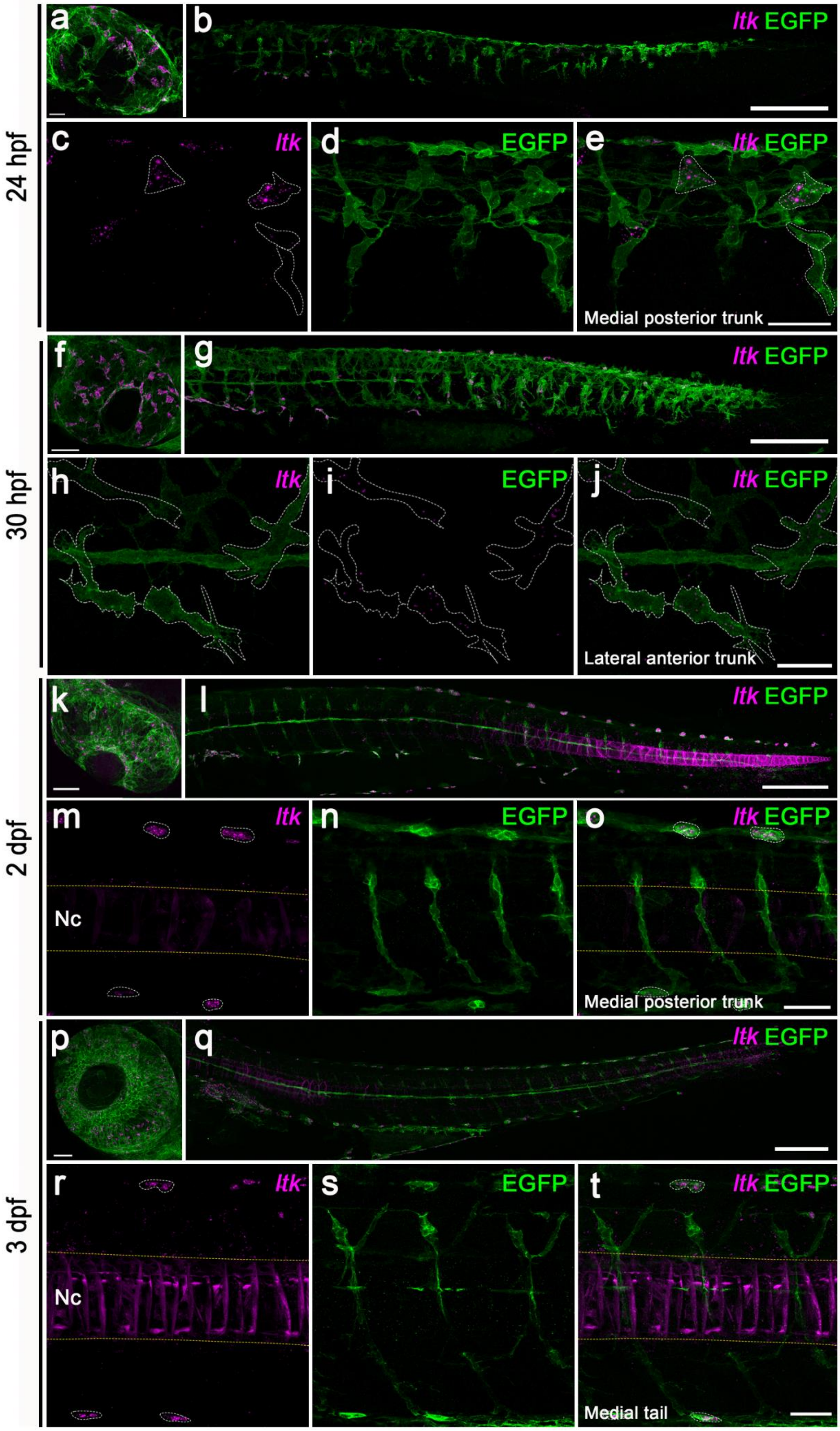

24 hpf
a
b
ltk EGFP
c
ltk
d
EGFP
e
ltk EGFP
Medial posterior trunk
30 hpf
f
g
ltk EGFP
h
ltk
i
EGFP
j
ltk EGFP
Lateral anterior trunk
2 dpf
k
l
ltk EGFP
m
ltk
Nc
n
EGFP
o
ltk EGFP
Medial posterior trunk
3 dpf
p
q
ltk EGFP
r
ltk
Nc
s
EGFP
t
ltk EGFP
Medial tail

Supplementary Figure 16 Detection of *ltk* transcripts during embryonic development by RNAscope. a-t,*Tg(Sox10:Cre)*^{ba74}*xTg(hsp70l:loxP-dsRed-loxP–Lyn-Egfp)*^{tud107Tg} embryos were heat shocked to induce enhanced GFP (GFP) expression, fixed at different stages and RNAscope detection of *ltk* transcripts was performed at 24 hpf (**a-e**), 30 hpf (**f-j**), 2 dpf (**k-o**) and 3 dpf (**p-t**). Expression in migrating NCC in medial pathway were observed first at 24 hpf (**c-e**), followed by cells migrating in the lateral pathway at 30hpf (**h-j**). Expression of *ltk* is mostly restricted to iridophores by 2 dpf (**m-o**) and 3 dpf (**r-t**). Note that autofluorescence is seen in the notochord (Nc, yellow dotted lines) from 2 dpf onwards. Dashed lines follow the cell membrane border as marked by GFP. Scale bar =200µm (**b, g, l,q**), 40µm (**c-e, m-o, r-t**) and 20µm (**h-j**).

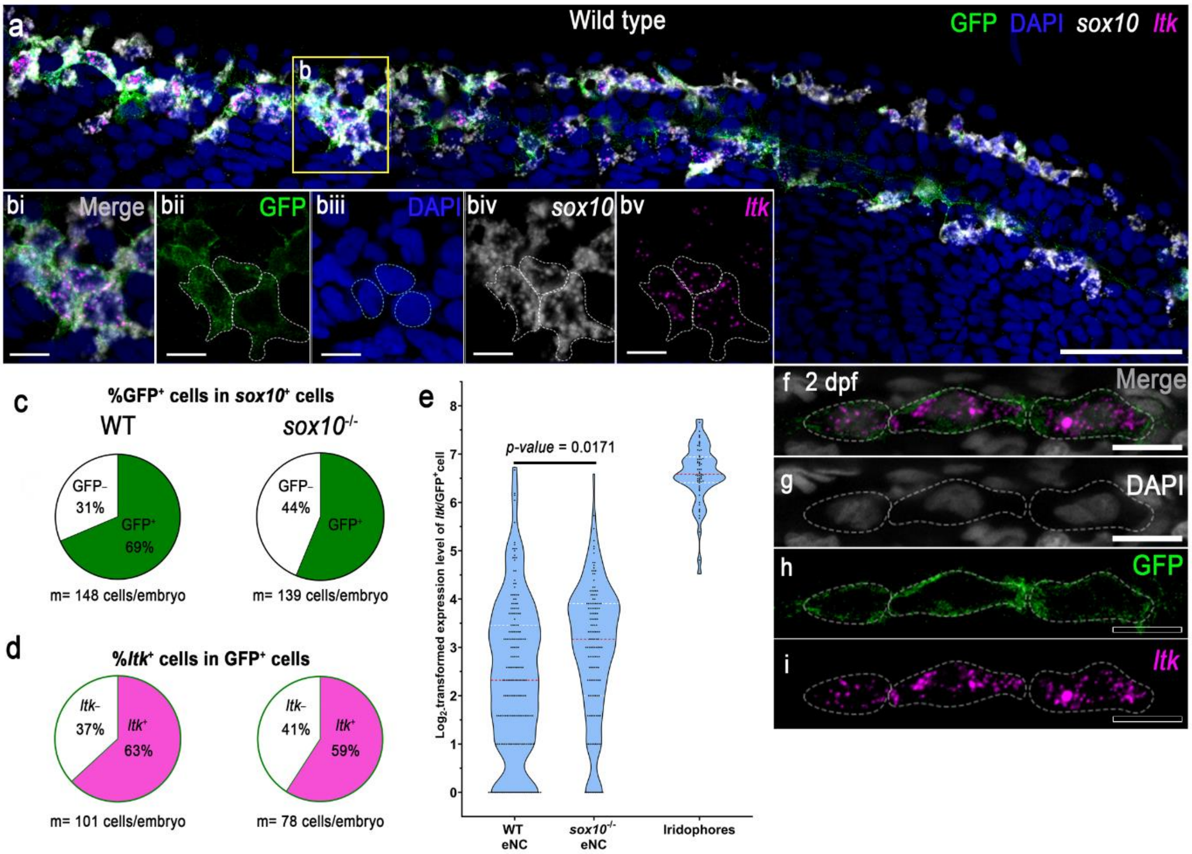

Supplementary Figure 17 Quantification of *ltk* expression by RNAscope supports Nanostring data. a-e, Expression of *ltk* in early NCCs in tail of WT embryos at 24 hpf (**a**); inset (**b**^{i}) shows example of three GFP-labelled cells (**b**^{ii}), their nuclei stained with DAPI (**b**^{iii}), expressing *sox10* (**b**^{iv}) and *ltk* (**b**^{v}). Quantification of mean percentage of GFP-positive cells in *sox10*-expressing cells in tails of WT and *sox10* mutants (*sox10*$^{-/-}$)(**c**) showed no significant difference (Two-tailed t-test, *p*-value=0.2717, n=3 embryos). Quantification of mean percentage of *ltk*-expressing cells amongst GFP-positive cells in tails of WT and *sox10*$^{-/-}$ mutants (**d**) showed no significant difference (Two-tailed *t*-test, *p*-value=0.5592, n=3 embryos). Quantification of absolute number of *ltk* transcripts in early NCCs of mutants shows a significantly increased number (log2 fold change of 0.313) in *sox10* mutants (*sox10*$^{-/-}$ eNC; **e**) compared to WT (WT eNC; **e**)(one-tailed test, *p-value*=0.0171, WT n=192 cells, *sox10*$^{-/-}$ n=140 cells examined in n=3 embryos per genotype). Pooled Log_2 transformed data is shown in violin plot (**e**). **f-i**, Examples of *ltk* expression in single iridophores at 2dpf. Quantification of numbers

of *ltk* transcripts in iridophores of 2 and 3 dpf WT embryos (Iridophores; **e**) shows an increase (log2 fold change of 1.5) compared to mean expression level in WT early NCCs. Note that all embryos analysed had *Tg*(*sox10:cre; hsp70I:loxp-dsred-STOP-loxp-LYN-EGFP*) transgene and were analysed at 24 hpf after brief heat-shock at 20 hpf to reveal GFP expression. Scale bars: 50 μm (a), 5 μm (b^{i}-b^{v}) and 10 μm (f-i). Source data are provided in Source Data file.

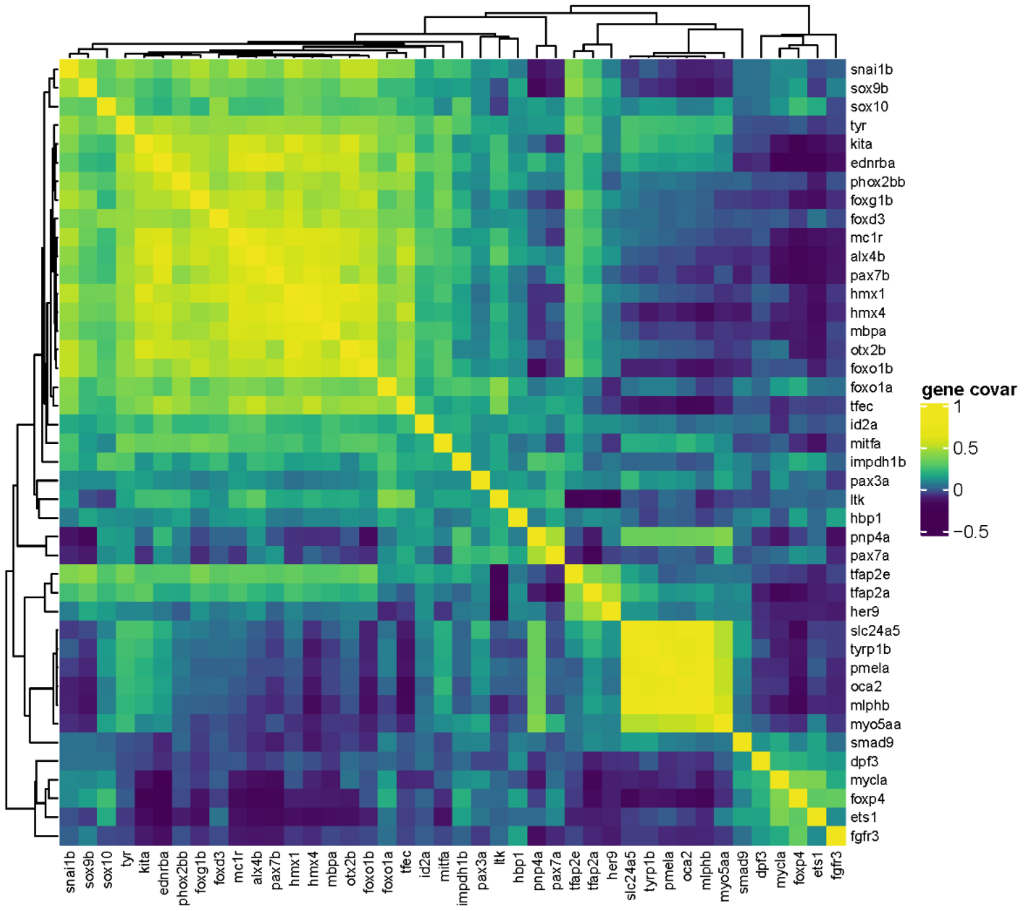

Supplementary Figure 18 Covariance of RNA counts for genes in WT cells.

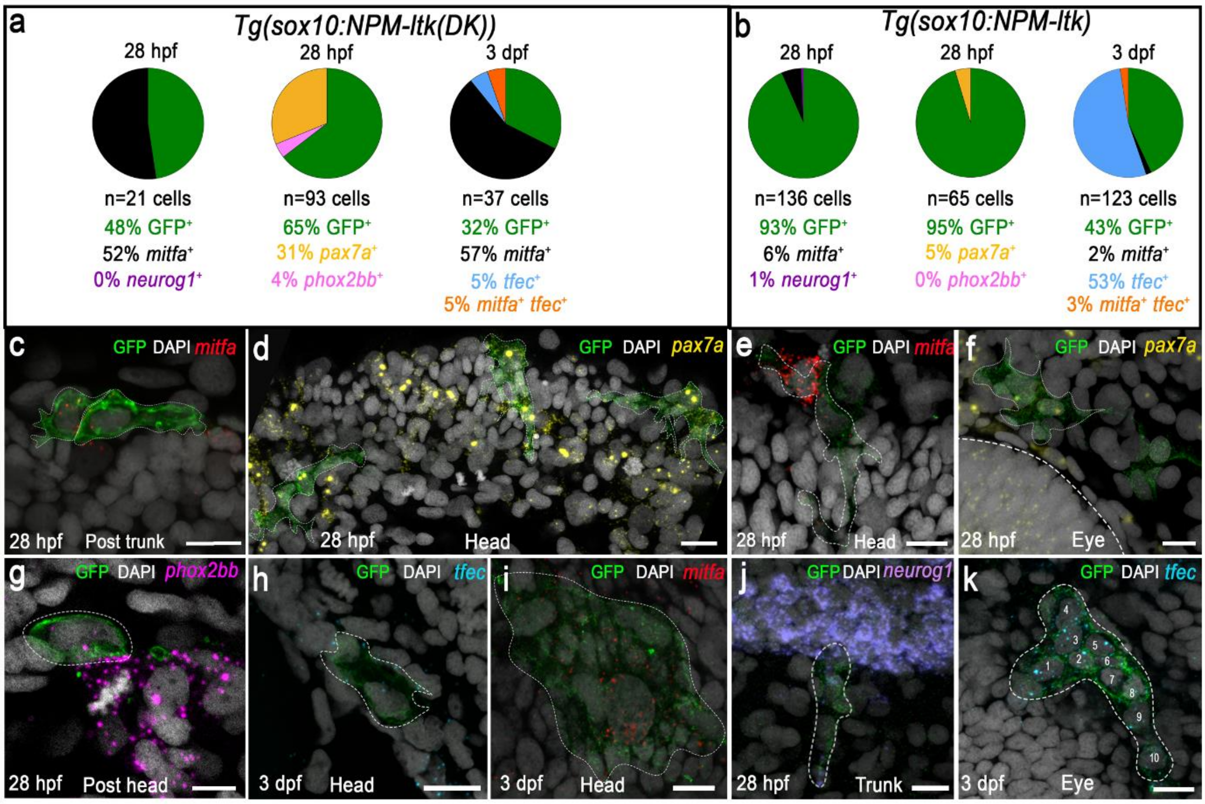

Supplementary Figure 19 Ltk signalling represses expression of non-iridophore fate specification transcription factors. **a,** Injection of *Tg(sox10:NPM-ltk(DK))* shows overlap of GFP-labelled cells (GFP+) with transcription factors *mitfa* and *nrgn1* or *pax7a* and *phox2bb* at 28hpf, and *mitfa* and *tfec* dorsally at 3 dpf. **b**, Injection of constitutively-activated form *Tg(sox10:NPM-ltk)* results in a reduction in the proportion of GFP-labelled cells expressing *mitfa, pax7a*, and *phox2bb* at 28 hpf. Correspondingly, the proportion of *tfec*-expressing cells found dorsally is strongly increased. **c-d,g-I**, Examples of GFP-labelled cells in embryos injected *Tg(sox10:NPM-ltk(DK))* expressing *mitfa* I, *pax7a* (**d**) and *phox2bb* (g) at 28 hpf, and *tfec* (h) and *mitfa* (i) at 3dpf. **e-f,j-k**, Examples of GFP-labelled cells in embryos injected with *Tg(sox10:NPM-ltk)* expressing *mitfa* (**e**), *pax7a* (**f**) and *nrgn1* (**j**) at 28 hpf and multiple *tfec*-expressing cells (**k**) at 3 dpf. Dashed lines follow the cell membrane marked by GFP. Scale bar =10 μm. Source data are provided in Source Data file.

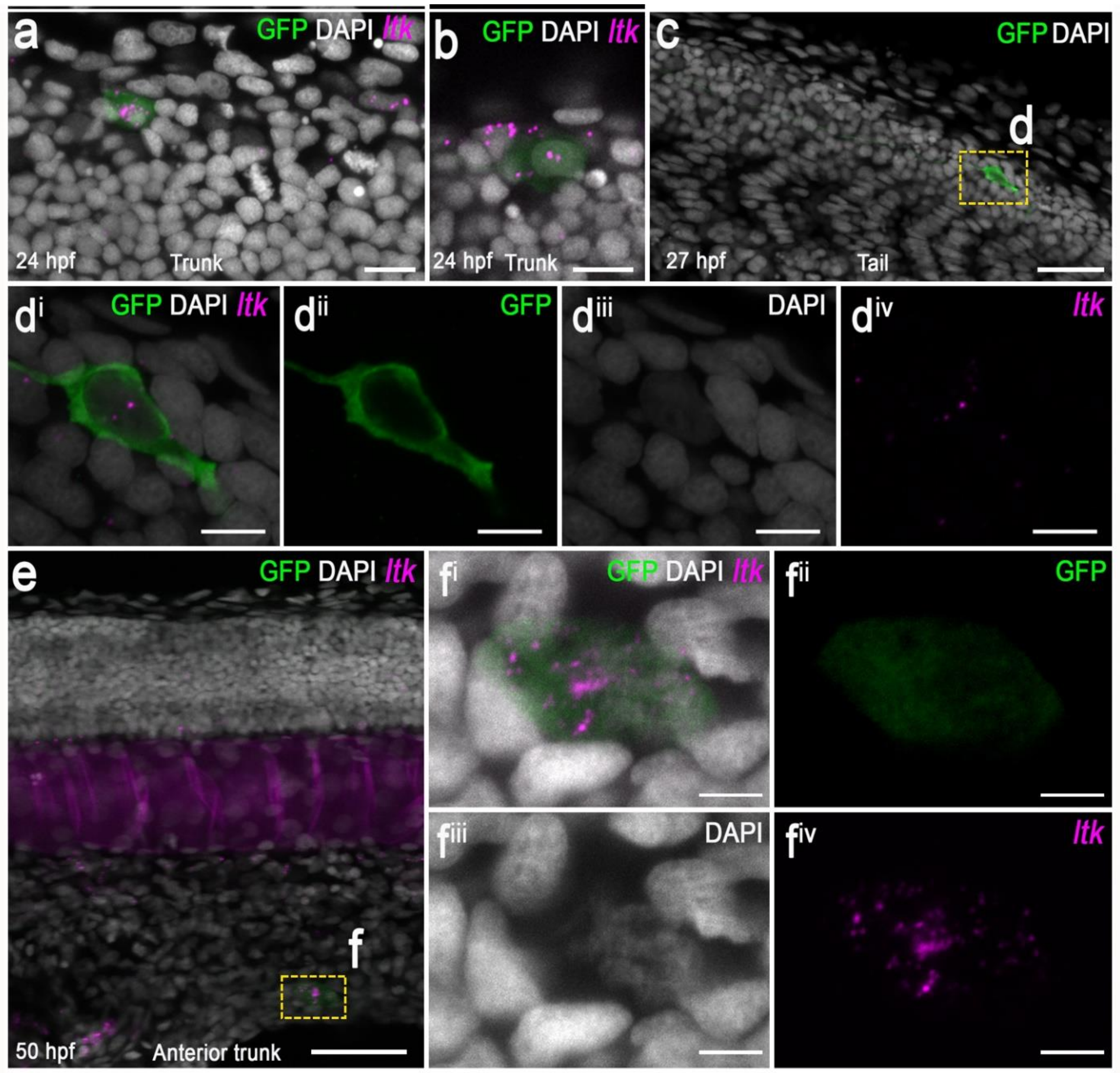

Supplementary Figure 20 GFP expression from *TgBAC(ltk:gfp)* follows *ltk* expression. **a-g^{iii}**, Injection of *TgBAC(ltk:gfp)* shows overlap of GFP labelled cells with *ltk* expression. **a-b**, Two examples of GFP-labelled cells in 24 hpf embryo in dorsal side of the trunk (a, b). **c-d^{iv}**, overview of tail of 27 hpf embryo shows GFP labelled cell (**c**), higher magnification imaging of inset (d) shows labelled cell with low expression of *ltk* (**d^{i}-d^{iv}**) in presumptive early NCC. **e-f^{iv}**, Embryo at 50 hpf showing GFP labelled cell in ventral stripe region (boxed, **f**); higher magnification imaging of inset (f) shows labelled cell with expression of *ltk* (**f^{i}-f^{iv}**), with morphology and location consistent with identity as iridophore. Scale bar =10µm (**a,b)**, 25µm (**c**), 25 µm (**e^{i}-e^{iv}**), 50µm (**e**) and 5µm (**f^{i}-f^{iv}**).

Supplementary References

1 Wagner, D. E. *et al.* Single-cell mapping of gene expression landscapes and lineage in the zebrafish embryo. *Science* **360**, 981-987, doi:10.1126/science.aar4362 (2018).

2 Saunders, L. M. *et al.* Thyroid hormone regulates distinct paths to maturation in pigment cell lineages. *Elife* **8**, doi:10.7554/eLife.45181 (2019).

3 Howard, A. G. t. *et al.* An atlas of neural crest lineages along the posterior developing zebrafish at single-cell resolution. *Elife* **10**, e60005, doi:10.7554/eLife.60005 (2021).

4 Lencer, E., Prekeris, R. & Artinger, K. B. Single-cell RNA analysis identifies pre-migratory neural crest cells expressing markers of differentiated derivatives. *Elife* **10**, doi:10.7554/eLife.66078 (2021).

5 Brombin, A. *et al.* Tfap2b specifies an embryonic melanocyte stem cell that retains adult multifate potential. *Cell Rep* **38**, 110234, doi:10.1016/j.celrep.2021.110234 (2022).

6 Kenny, C. *et al.* TFAP2 paralogs facilitate chromatin access for MITF at pigmentation and cell proliferation genes. *PLoS Genet* **18**, e1010207, doi:10.1371/journal.pgen.1010207 (2022).

7 Petratou, K. *et al.* A systems biology approach uncovers the core gene regulatory network governing iridophore fate choice from the neural crest. *PLoS Genet* **14**, e1007402, doi:10.1371/journal.pgen.1007402 (2018).
